# Artificial intelligence for stromal cell quality assessment: A systematic review of human mesenchymal stromal cell properties and viability

**DOI:** 10.1007/s00441-026-04097-y

**Published:** 2026-10-15

**Authors:** William Li, Himal Kandel, Stephanie Baker, J. Guy Lyons, Mojdeh Abbasi, Shrushti Surya, Athanasia Vardakis, Yuri Gervasiev, Stephanie Watson

**Affiliations:** 1 Sydney Medical School, Faculty of Medicine and Health, The University of Sydney, Sydney, New South Wales Australia; 2 College of Science and Engineering, Faculty of Engineering, James Cook University, Cairns, Queensland Australia; 3 Centenary Institute, The University of Sydney, Missenden Rd, Camperdown, New South Wales 2050 Australia; 4 Department of Dermatology, The University of Sydney at Royal Prince Alfred Hospital, Missenden Rd, Camperdown, Sydney, New South Wales 2050 Australia

**Keywords:** Artificial intelligence, Mesenchymal stromal cells, Stemness, Viability

## Abstract

Reliable assessment of mesenchymal stromal cell (MSC) viability and stem cell–associated properties is essential for ensuring the consistency, safety, and therapeutic potential of MSC cultures before clinical use. However, incomplete understanding of the mechanisms underlying MSC therapeutic effects remains a major barrier to clinical translation and to establishing reliable, standardised quality attributes. Artificial intelligence (AI) may enable automated assessment of these properties, but the performance and clinical relevance of AI-based approaches have not been systematically evaluated. This systematic review evaluated the accuracy and clinical utility of AI for assessing viability and stem cell–associated properties in human MSC cultures. Following PRISMA guidelines, we searched five electronic databases (Cochrane, Scopus, PubMed, EMBASE, and MEDLINE) for eligible studies reporting quantitative AI-based assessments of human MSC cultures. Two reviewers independently screened studies, extracted data, and assessed risk of bias using PROBAST. The review protocol was prospectively registered with PROSPERO (CRD420256393235). Of 3,872 records identified, 18 studies met the inclusion criteria. Supervised AI models, particularly convolutional neural networks, were most used. Across included studies, reported performance metrics varied substantially because of differences in AI models, imaging modalities, datasets, and outcome definitions. Considerable heterogeneity in AI model architectures, imaging modalities, outcome definitions, and reporting metrics limited direct comparison across studies. Overall, available evidence suggests that AI can assess key MSC quality attributes and may support more objective, scalable, and standardised quality-control workflows. However, broader validation, standardised reporting, external testing, and evaluation across a wider range of MSC characteristics are required before routine clinical implementation can be recommended.

**Supplementary information:**

The online version contains supplementary material available at https://doi.org/10.1007/s00441-026-04097-y.

## Introduction

Mesenchymal stromal cells (MSCs) play essential roles in regeneration and repair within the body (Chen et al. 2023; Wang et al. 2025a). MSCs possess immunomodulatory and multipotent differentiation capabilities (Zhu et al. 2025b) and have roles such as in corneal stromal repair and regeneration (El Zarif et al. 2021; Oh et al. 2008; Watson et al. 2010). Mesenchymal stromal cells are attractive candidates for regenerative therapies. Effective clinical translation of stem cell-based therapies requires robust quality control of cultured cells, particularly with respect to viability and maintenance of stemness.

Conventional methods for assessing stem cell cultures rely on manual techniques such as morphological evaluation, biomarker staining, and colony-forming assays (Chen et al. 2023). These approaches are time-consuming, resource-intensive, subject to interobserver variability, and limited in scalability (Chabert et al. 2018). Recent advancements in artificial intelligence (AI), including machine learning and deep learning, offers potential solutions by enabling objective, high-throughput analysis of stem cell characteristics (Mota et al. 2021). There remains limited consolidated evidence on the application and outcomes of AI in regenerative medicine.

This systematic review aimed to (1) systematically summarise artificial intelligence methods used to assess stemness and viability in human MSCs, (2) synthesise the reported performance of these models where appropriate, and (3) critically evaluate their current readiness for stem cell quality assessment and future clinical translation. We synthesized existing studies on AI applications in MSC culture evaluation to identify the types of AI architectures employed, input features analysed, and performance outcomes reported. By mapping the current landscape, this review identifies current evidence gaps and may inform future efforts to standardize AI-based stem cell quality assessment for regenerative medicine. Here, mapping refers to systematically characterising and organising the available evidence according to study characteristics, including MSC source, imaging modality, AI architecture, input features, target outcome, reference standard, and reported performance.

For the purposes of this review, stemness refers to the capacity of MSCs to maintain self-renewal and multipotent differentiation potential while retaining their characteristic biological phenotype. As no single biomarker or functional assay fully defines MSC stemness, the included studies evaluated surrogate indicators including differentiation capacity, senescence status, morphology, proliferation, gene or protein expression, metabolic activity, and other imaging-derived features. Viability refers to the proportion of living, healthy cells capable of maintaining membrane integrity and normal metabolic function. Accordingly, studies assessing viability through morphological analysis, cell segmentation, or other image-based measures of live cell status were considered eligible. These definitions were used to provide a consistent framework for interpreting the diverse AI applications identified in this review. Notably, Silva Couto et al. conducted a side-by-side comparison of adult- and perinatal-derived hMSCs, demonstrating source-dependent biological differences that may affect manufacturing processes and the generalisability of AI models across MSC sources (Silva Couto et al. 2024).

## Materials and methods

**Fig. 1:**
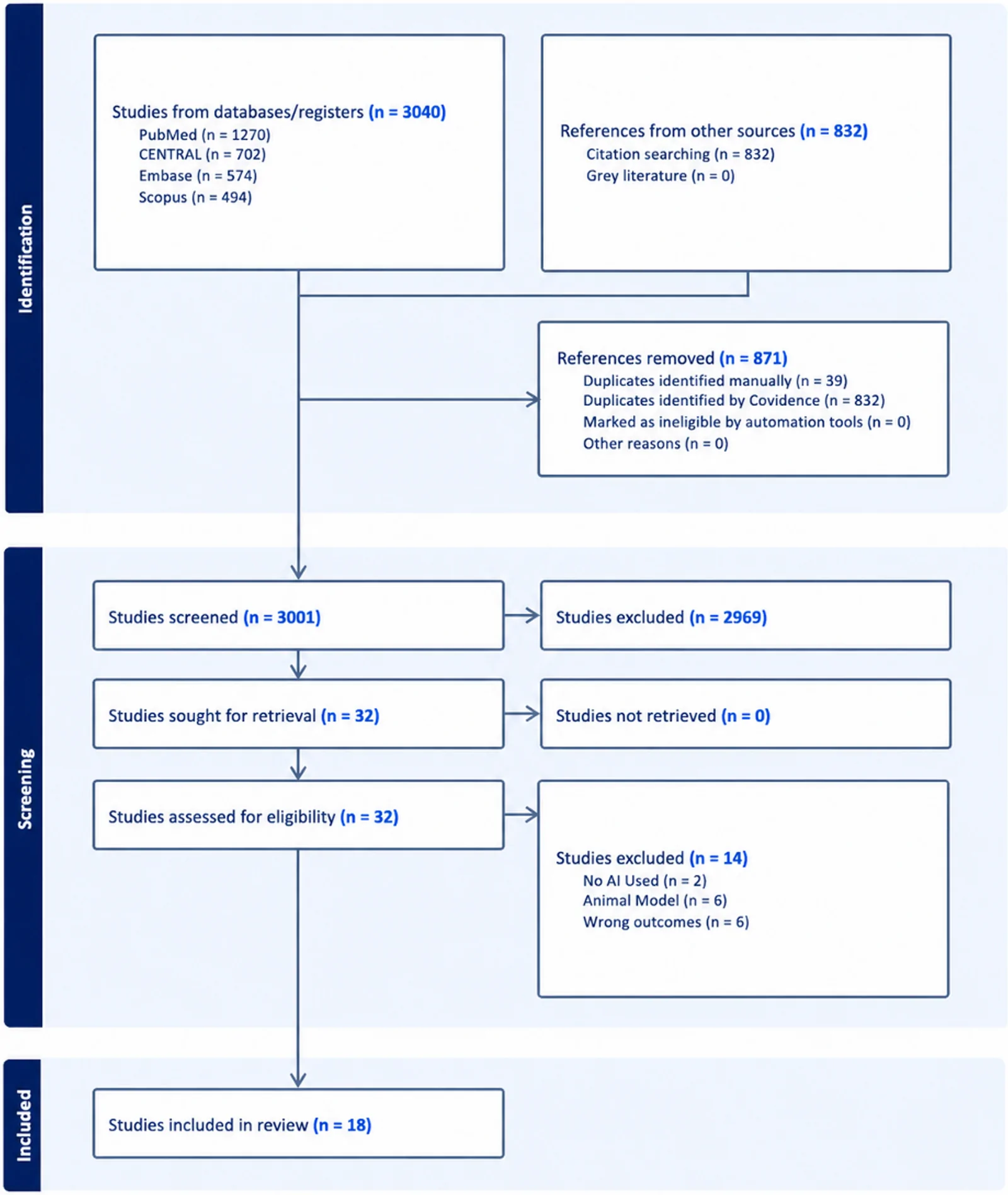
PRISMA Flow Diagram: After database and citation searching, duplicates were removed, and records screened. Following full-text review, 18 studies met eligibility criteria and were included

This systematic review was conducted in accordance with the Preferred Reporting Items for Systematic Reviews and Meta-Analyses (PRISMA) guidelines (Page et al. 2021) (Fig. 1). The review protocol was prospectively registered with the International Prospective Register of Systematic Reviews (PROSPERO; Registration No. CRD42025639235)

### Search strategy

A comprehensive literature search was conducted across multiple electronic databases, including Cochrane, Scopus, PubMed, EMBASE, and MEDLINE from the 3rd of January 2025 to the 11th of April 2025 (Appendix A). Searches were performed using database-specific combinations of title, abstract, keyword, and controlled vocabulary fields, as appropriate for each database. The search was limited to publications from the last 10 years, with additional searches in the Clinical Trials.Gov database for preliminary or ongoing studies.

The search strategy combined MeSH terms, keywords, and Boolean operators as highlighted in Table 1. Key concepts included: 1) Artificial intelligence (AI, machine learning, deep learning, neural networks) 2) Stem cell culture (MSC culture, optimization, regenerative medicine) and 3) Biomarkers and cell viability (stemness markers, proliferation, multipotency, differentiation potential). The search included limbal epithelial stem cells (LESC) but no studies were found on this topic.

**Table 1:** List of keywords and MeSH terms used to identify relevant studies on AI-based assessment of mesenchymal and limbal epithelial stem cell cultures

	Domain- specific terms	Methodological terms
Keywords	Stem cell* - Mesenchymal stromal cell* - Limbal epithelial stem cells* - Ocular therapies - Regenerative medicine - Stemness - Differentiation capacity - Proliferation - Self-renewal - Multipotency - Transcription factors - Senescence - Viability - Trypan blue - Live/Dead staining	- Machine learning - Neural network* - Supervised learning - Deep learning
MeSH terms	- Artificial intelligence	- Machine learning - Unsupervised machine learning

### Inclusion and exclusion criteria

Eligible studies were original research articles evaluating AI applications in human MSC cultures, published within the last ten years, that reported quantitative outcomes related to MSC stemness and/or viability. We accepted studies whether they used newly collected data or existing datasets, provided that the analysis was original. Eligible studies were required to present original data and report quantitative outcomes related to MSC stemness and/or cell viability. Studies were excluded if they were of a different design (e.g., reviews, editorials, conference abstracts, or letters) or did not report culture-related outcome measures.

For this review, culture characterisation was defined as assessment of MSC attributes relevant to cell quality, including stemness-associated properties, viability, morphology, proliferation, senescence, differentiation capacity, and molecular markers. Manufacturing, process optimisation, and dose optimisation were not considered independent eligibility domains unless they included a quantitative AI-based assessment of one or more of these culture-characterisation outcomes.

AI/ML involvement was defined as the use of computational models that learn patterns from data to classify, predict, segment, detect, or quantify MSC culture characteristics. Eligible studies were required to apply AI/ML directly to culture-derived data, including microscopy images, molecular data, or other quantitative culture measurements, to assess stemness- or viability-related outcomes.

### Screening

After title and abstract screening, 32 studies underwent full-text review, of which 16 met the inclusion criteria following the primary database search. Reference screening of the included studies identified 2 additional eligible studies, resulting in a total of 18 included studies as shown in Fig. 1.

Abstract and full-text screening were independently conducted by two reviewers (WL, SS, YG, AV), with each reviewer assigned a specific subset of papers to screen. Discrepancies were resolved through discussion or when necessary, resolved by a third reviewer who was not involved in the initial screening (WL, SS, YG AV). Risk of bias was assessed using the Prediction model Risk Of Bias Assessment Tool (PROBAST). Although PROBAST was originally developed for diagnostic and prognostic prediction model studies, it was selected because the included studies primarily developed or validated AI prediction models reporting performance metrics such as accuracy, sensitivity, specificity, precision, F1 score, and area under the receiver operating characteristic curve (AUC). While several studies involved image segmentation or computer vision tasks, their primary objective remained predictive model development or evaluation, making PROBAST the most appropriate available framework for a consistent assessment of methodological quality across studies.

A total of 23 key variables were extracted from each study, including:

1. **Study details:** Title, first author’s name, year of publication, country of study, and study aim.
2. **AI model information:** Type of AI model used, number of models used, and training approach.
3. **Stem cell focus:** Type of stem cells studied, stemness/viability markers targeted such as SOX2, OCT4, NANOG, senescence, morphology, proliferation, and differentiation.
4. **Performance metrics:** Accuracy, Pearson correlation coefficient (PCC), sensitivity, specificity, Area under the curve (AUC), F1 score, precision, dice similarity coefficient (DSC), Harsdorf distance (HD), intraclass correlation coefficient (ICC), variance explained estimate (VEE), segmentation (SEG) score, and leave-one-out cross-validated r-squared (LOO-R^2^) score.

The primary outcomes were AI performance metrics for assessing stemness and viability in MSC cultures. Markers included colony-forming efficiency, senescence, transcription factors (e.g., SOX2, OCT4, NANOG), morphology, proliferation, senescence, and differentiation capacity. Secondary outcomes included AI model type and training approach (e.g., supervised, convolutional neural network).

### Data synthesis

Extracted study characteristics and AI performance metrics were summarised descriptively in tables and narrative form. Reported metrics were organised by outcome type (stemness or viability) and metric type, including sensitivity, specificity, accuracy, precision, F1 score, AUC, and correlation or segmentation measures. As studies differed substantially in AI architectures, imaging modalities, datasets, outcome definitions, and reported metrics, a formal meta-analysis was not undertaken.

### Risk of bias

For each study, each domain of Prediction model Risk Of Bias Assessment TOOL (PROBAST)—selection, predictors, outcome, analysis, overall risk of bias was evaluated using PROBAST criteria (Wolff et al. 2019).

## Results

The number of publications applying AI to MSC stemness and viability has increased in recent years. Of the 18 studies included, 1 was published in 2017, 1 in 2018, 3 in 2021, 2 in 2022, 4 in 2023, 4 in 2024 and 3 in 2025 as detailed in Table 2.

**Table 2:** Characteristics of all articles included in AI assessment of mesenchymal stem cell viability and stemness

Study author and year	AI model type	Learning task	Assessed parameter	Measured outcome (%)	Sample size of the testing set (n of images provided to the AI model)	Comments, reason for exclusion from quantitative analysis	MSC tissue source
Kong 2023 (Kong et al. 2023)	1 × Unsupervised K-means	Clustering	Stemness via differentiation status of MSCs to adipogenic or osteogenic lineages from microscopic images and raman scattering for lipid content	Cluster proportion of differentiated cells	175	Qualitative data was reported	Not specified
Mota 2021 (Mota et al. 2021)	5 × Supervised linear SVM, radial-basis SVM, LDA, KNN, LR	Classification	Viability via segmentation of MSCs and classification their phenotype using phase contrast micrographs of monolayer culture	Segmentation: Sensitivity: 88 Precision: 86 DSC: 84.9 Classification: AUC: 81.6, 78.7 Sensitivity: 78.9, 88.7, 79.6 Specificity: 75.7	36	N/A	Bone marrow
Ghabriel 2023 (Ghabrie et al. 2023)	1 × Supervised RF	Classification	Stemness via gene expression analysis of 6 proteasome genes associated with stemness	Accuracy: 100	N/A	mRNA levels of 22 proteasome genes were measured rather than images	Umbilical cord, amnion, bone marrow and adipose tissue
Cáelebi 2024 (Çelebi et al. 2024)	4 × Self-supervised learning and then supervised fine-tuning CNN	Segmentation	Stemness via segmentation and identification of senescent MSCs	Precision: 21.6, 35.1, 39.3, 55.9, 64.8, 65, 65.3, 73.6, 74.2, 81.9 Sensitivity: 33, 50.8, 69.4, 71, 73.5, 73.8, 78.5, 81.9, 83.2, 92.8 DSC: 63.6, 73.9, 97, 100	30	N/A	Adipose tissue
Wang 2025 (Wang et al. 2025a)	4 × Unsupervised PCA followed by supervised SVM, LDA, and DFA	Clustering and classification	Both via ageing, heterogeneity, and biochemical composition changes with passage	Accuracy: 77.2, 76.7, 44.6, 52.2 Sensitivity: 44.6, 52, 76.2, 76.8 Specificity: 93.1, 94, 97.1, 97.2	N/A	Spectroscopy was measured rather than images	Umbilical cord
He 2024 (He et al. 2024)	8 × Supervised cascade R-CNN	Classification	Stemness via senescence status of MSCs	Precision: 81, 85, 87.4 Sensitivity: 81.4, 92.3, 93, 93.4 F1: 88.5, 90.3	807	N/A	Peripheral blood mononuclear cells
Xiao 2022 (Xiao et al. 2022)	6 × Supervised transformer	Classification	Stemness via senescence status of MSCs	Precision: 60.6, 60.8, 65.2, 66.8, 72.1, 80, 78.4, 78.1, 70.6, 75.8, 89.5, 91.6 Sensitivity: 89.6, 93.2, 95.6, 93.2, 93.1, 91.7, 88.8, 86.1, 89.3, 91.4, 91.4, 80 F1 score: 84.5, 85.2, 86, 80.4, 85.2, 91.7, 79.6, 71.1, 75.4, 73, 77.2, 84.5	N/A	Testing set data not given	iPSCs of non-specific origin
Vega 2017 (Vega et al. 2017)	1 × Supervised SVM and unsupervised PCA	Classification	Stemness via MSC images, telomerase expression and cell surface markers	Sensitivity: 86.8, 94.7 Specificity: 80.7, 91.7	N/A	Testing set data not given	Bone marrow
Zhu 2025 (Zhu et al. 2025a)	1 × Supervised CNN	Classification and regression	Both via Raman spectroscopy and microscope images to cell count, assess confluence, and biochemical changes	Accuracy: 93 F1 score: 93	150	N/A	Umbilical cord
Imai 2018 (Imai et al. 2018)	3 × Unsupervised PCA and supervised LR and LDM	Classification and regression	Viability via morphology-based image analysis	Accuracy: 90.7	330	N/A	Bone marrow and adipose tissue
Kim 2022 (Kim et al. 2022b)	4 × Supervised CNN	Classification	Stemness via predication of multipotency from morphology	Accuracy: 82.6, 83.2, 83.2, 83.8 Sensitivity: 97.5, 98.7, 98.6, 99.1 Specificity: 63.6, 63.6, 63.6, 64.3 F1 score: 86.2, 86.8, 86.8, 87.2 AUC: 87.4, 89.1, 84.4, 86.1	370	N/A	Nasal turbinate
Imboden 2021 (Imboden et al. 2021)	2 × Supervised CNN trained inside of cGAN	Regression	Stemness via quantifying heterogeneity by predicting 8 protein markers expression to capture heterogeneity	PCC: 77	300	N/A	Bone marrow
Shi 2024 (Shi et al. 2024)	6 × Supervised CNN	Classification	Stemness via differentiation status of MSCs, allowing detection of stemness loss within 1 day	Accuracy: 82.7 AUC: 85.3 Precision: 78.5 Sensitivity: 81.2 F1 score: 77.9	60	N/A	Umbilical cord
VanGrouw 2023 (Van Grouw et al. 2023)	7 × Supervised SR, LASSO, DT, RF, SVM, PLSR, and GBM	Regression	Stemness via metabolic markers predicative of MSC potency	LOOR score: 98.5, 82, 87.8, 85.8, 77, 71.5, 78.9	N/A	Spectroscopy was measured rather than images	Bone marrow and induced pluripotent stem cells
Kim (2) 2022 (Kim et al. 2022a)	5 × Supervised CNN	Classification	Stemness via classification of MSCs into either high or low multilineage-differentiating stress enduring cells (MUSE) cell marker groups via microscopic images	AUC: 88.5, 89.3, 94.1, 92.7, 90.1 F1: 76.8, 76.9, 89.3, 86.5, 81.2 Accuracy: 79.3, 79.2, 89.2, 85.8, 82.7 Sensitivity: 68.9, 86.7, 89.9, 83, 90 Specificity: 89.7, 86.7, 89.9, 83, 88.6	12,240	N/A	Nasal turbinate
Wu 2025 (Wu et al. 2025)	5 × Supervised CNN	Classification	Stemness via stiffness of MSCs	AUC: 100 F1: 97.6 Accuracy: 97.6 Precision: 96.8 Sensitivity: 98.4	24,000	N/A	Adipose tissue, bone marrow and umbilical cord
Weber 2023 (Weber et al. 2023)	1 × Supervised cGAN	Regression	Stemness via detection of senescence markers including SABG, p16, p21 and p38	PCC: 80% for p16, 70% for p21	75	N/A	Adipose tissue and bone marrow
Carnevali 2024 (Carnevali et al. 2024)	Supervised CNN	Classification	Stemness via analyses of histone H3, RNA polymerase II, and DNA to identify distinct cell states	Accuracy: 85, 80, 87, 91, 94, 83 F1: 85, 80, 91 AUC: 95, 95, 97, 98, 95, 95, 89 Precision: 91, 86	455	N/A	Umbilical

Key: *N/A* not applicable, *SVM* support vector machine, *LDA* linear discrimination analysis, *KNN* k-nearest neighbour, *LR* logistical regression, *RF* random forest, *CNN* convolutional neural network, *DFA* discriminant function analysis, *PCA* principle component analysis, *cGAN* conditional generative adversarial network, *SR* step wise regression, *LASSO* least absolute shrinkage and selection operator, *DT* decision tree, *GBM*  gradient boosting machine, *PLSR* partial least square regression, *iPSC* induced pluripotent stem cell

### Countries of published studies on AI in measuring MSC viability and/or stemness

The included studies were primarily conducted in a small number of countries, with China (*n* = 7 publications) and the USA (*n* = 6) leading the research output. Other contributing countries included Korea (*n* = 2), Egypt (*n* = 1), Turkey (*n* = 1), and Japan (*n* = 1).

### Type of AI models used

Seventeen of the 18 included studies used supervised models, most commonly CNNs (Carnevali et al. 2024; Çelebi et al. 2024; Imboden et al. 2021; Kim et al. 2022a; Shi et al. 2024; Wu et al. 2025; Zhu et al. 2025a) and SVMs (Mota et al. 2021; Van Grouw et al. 2023; Vega et al. 2017; Wang et al. 2025a). Random Forests were less frequent (Ghabriel et al. 2023; Van Grouw et al. 2023), alongside others such as LDA (Mota et al. 2021; Wang et al. 2025a), KNN (Mota et al. 2021), LR (Imai et al. 2018; Mota et al. 2021), cascade R-CNN (He et al. 2024), transformers (Xiao et al. 2022), DFA (Wang et al. 2025a), SR, LASSO, DT, PLSR, GBM (Van Grouw et al. 2023), LDM (Imai et al. 2018), and cGANs (Imboden et al. 2021; Weber et al. 2023). Five studies which used unsupervised methods: K-means (Kong et al. 2023), PCA (Imai et al. 2018; Vega et al. 2017; Wang et al. 2025a), and self-supervised pre-training (Çelebi et al. 2024). Some studies (Çelebi et al. 2024; Imai et al. 2018; Vega et al. 2017; Wang et al. 2025a) combined supervised and unsupervised approaches.

### Type of stem cells measured in studies

All 18 studies in this review studied MSCs. Most studies (*n* = 12) (Çelebi et al. 2024; He et al. 2024, Imai et al. 2018; Imboden et al. 2021; Kim et al. 2022a; Kong et al. 2023; Mota et al. 2021; Shi et al. 2024; Van Grouw et al. 2023; Vega et al. 2017; Wang et al. 2025a; Wu et al. 2025) used bone-marrow–derived MSCs. Four studies (Carnevali et al. 2024; Kim et al. 2022b; Weber et al. 2023; Zhu et al. 2025a) used adipose-derived MSCs. One study sourced their MSCs from multiple tissue sources such as bone marrow, adipose, umbilical cord, and amnion (Ghabriel, El Hosseiny, Moustafa and Amleh, 2023).

### Whether viability or stemness was measured in studies

Stemness was assessed using differentiation into adipogenic or osteogenic lineages (Kong et al. 2023), gene expression of proteasome genes (Ghabriel et al. 2023), and senescence markers (Çelebi et al. 2024; He et al. 2024; Weber et al. 2023; Xiao et al. 2022). Other methods included MSC imaging with telomerase and surface markers (Vega et al. 2017), analysis of histone H3, RNA polymerase II, and DNA (Carnevali et al. 2024), prediction of multipotency from morphology (Kim et al. 2022b), classification by MUSE markers (Kim et al. 2022a), prediction of protein marker expression (Imboden et al. 2021), early detection of stemness loss via differentiation status (Shi et al. 2024), metabolic markers (Van Grouw et al. 2023), and cell membrane stiffness (Wu et al. 2025).

Viability was assessed using segmentation and classification of MSCs in phase contrast micrographs (Mota et al. 2021) and morphology-based image analysis (Imai et al. 2018).

Stemness and viability were assessed through ageing, heterogeneity, and biochemical changes using spectroscopy (Wang et al. 2025a).Raman spectroscopy with microscopy was also used to evaluate cell count, confluence, and biochemical changes (Zhu et al. 2025a).

### Performance metrics detail in AI models

**Fig. 2:**
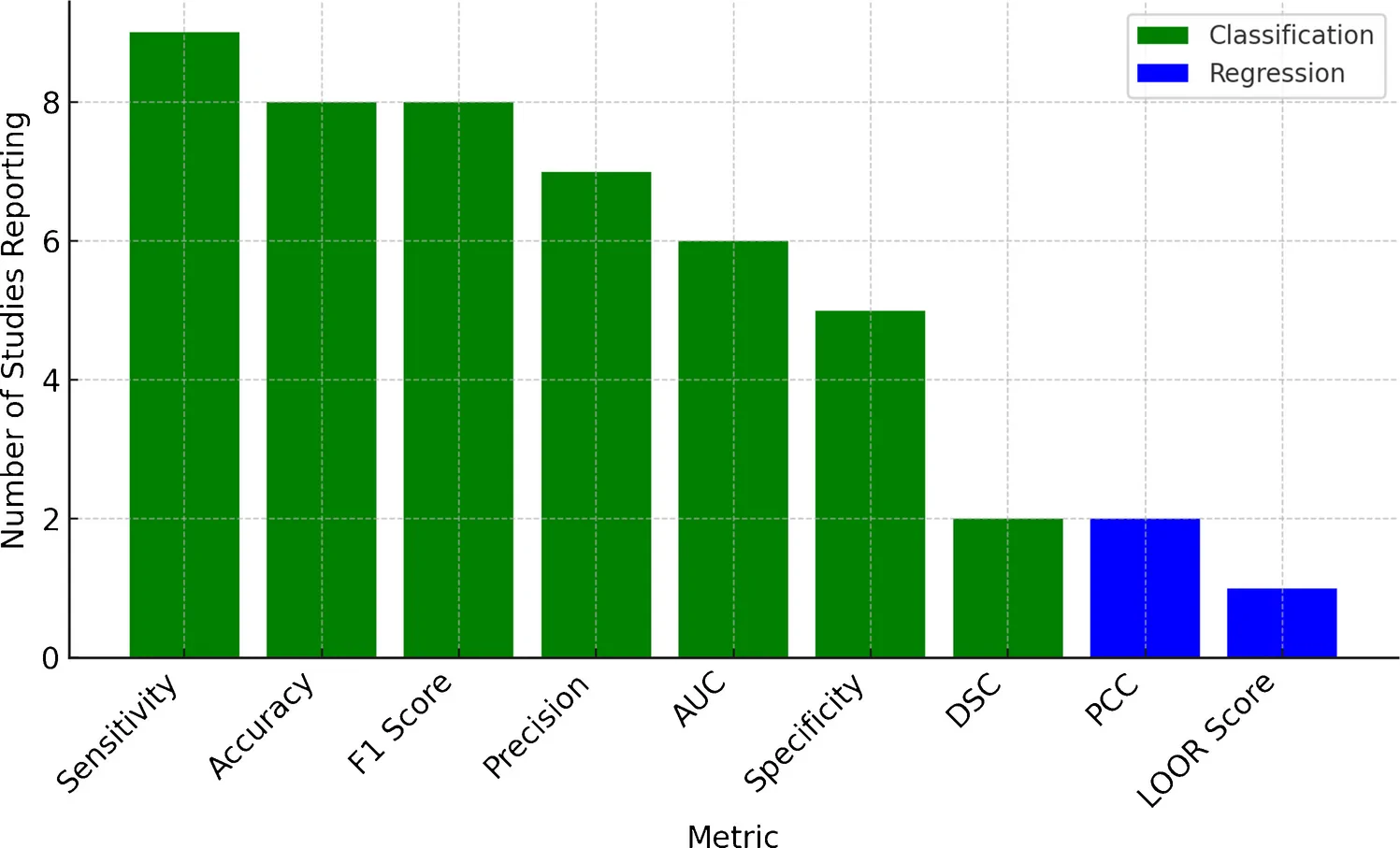
Frequency of reported AI performance metrics across all included studies. Metrics include sensitivity, accuracy, F1 score, precision, specificity, and overlap/correlation measures (AUC = area under the curve; DSC = dice similarity coefficient; PCC = Pearson correlation coefficient; LOO-R^2^ = leave-one-out regression score)

Sensitivity was the most frequently reported performance metric, followed by accuracy, F1 score, precision, and AUC (Çelebi et al. 2024; He et al. 2024; Kim et al. 2022b; Mota et al. 2021; Shi et al. 2024; Vega et al. 2017; Wang et al. 2025a; Wu et al. 2025; Xiao et al. 2022). Accuracy and F1 score were also frequently used (Carnevali et al. 2024; Ghabriel et al. 2023; He et al. 2024; Imai et al. 2018; Kim et al. 2022a, 2022b; Shi et al. 2024; Wang et al. 2025a; Wu et al. 2025; Xiao et al. 2022; Zhu et al. 2025a). Precision and AUC were less common (Carnevali et al. 2024; Çelebi et al. 2024; He et al. 2024; Kim et al. 2022a, 2022b; Mota et al. 2021; Shi et al. 2024; Wu et al. 2025; Xiao et al. 2022). Specificity appeared in fewer studies (Kim et al. 2022a, 2022b; Mota et al. 2021; Vega et al. 2017; Wang, et al. 2025b). Overlap and correlation metrics such as DSC and PCC were rarely reported (Çelebi et al. 2024; Imboden et al. 2021; Mota et al. 2021; Weber et al. 2023). Only one study reported a LOO-R^2^ score (Van Grouw et al. 2023). Figure 2 illustrates the relative frequency of each performance metric reported across the included studies, highlighting sensitivity, accuracy, and F1 score as the most consistently used measures.

### Bias assessment

**Fig. 3:**
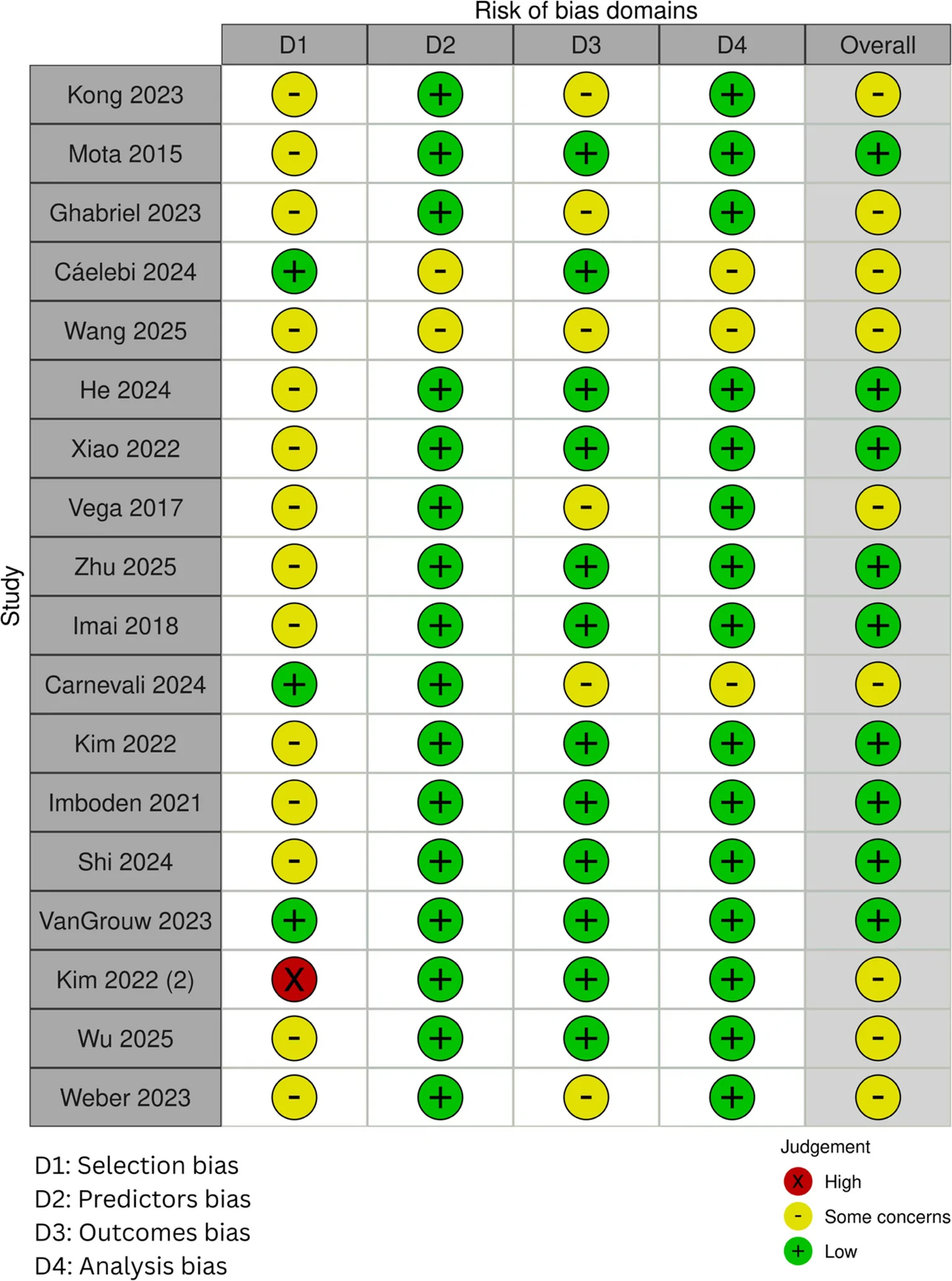
PROBAST risk assessment of all 18 included studies in a traffic light plot

Using the PROBAST tool, most studies showed either low risk or some concerns of bias. The main issues related to participant selection such as variation in the types of MSCs collected and outcome measurement as there was inconsistent evaluation of performance metrics. Overall, the evidence was moderately reliable but should be interpreted with caution given the heterogeneity in AI models, stem cells assessed, reported metrics, and outcome definitions (Fig. 3).

## Discussion

Most studies showed low to moderate risk of bias, suggesting the findings are generally reliable but should be interpreted with some caution. A key translational bottleneck is that the mechanisms through which hMSCs exert therapeutic effects remain incompletely understood. This uncertainty makes it difficult to define biologically meaningful potency assays and quality attributes, and therefore to determine whether AI-derived measures of morphology, viability, or stemness reliably predict clinical activity.

The growing number of publications in recent years suggests rising interest in AI-driven stem cell technologies from 1 study in 2017 to more than 3 a year from 2021 onwards (Monson et al. 2024). Most of the studies had been conducted in China or the USA. Supervised models, most commonly CNNs and SVMs were used in almost all studies (17 out of 18). A broad range of MSC sources was represented, predominantly bone-marrow (*n* = 12) and adipose-derived MSCs (*n* = 4), with a small number of studies using umbilical-cord or amnion-derived MSCs (*n* = 1). MSCs have broad potential for regenerative medicine in applications across the body (Chabert et al. 2018), which may explain the strong interest in this area. Most studies assessed morphology, senescence, differentiation capacity, or other surrogate indicators of MSC stemness, while a smaller number evaluated molecular markers.

Our findings suggest that AI could support more consistent MSC quality control by reducing observer-dependent variation and enabling scalable analysis of cell cultures. AI-based image analysis may help identify changes in viability and stemness-associated features earlier and more reproducibly than manual assessment alone. This could streamline culture monitoring and batch-release decision-making; however, AI outputs must be validated against biologically meaningful functional and potency assays before they can be used in clinical manufacturing (Ghabriel et al. 2023). Previous work has also shown that convolutional neural networks can automate cell detection, segmentation, and morphology-based classification from microscopy images, reducing reliance on manual cell counting while maintaining comparable performance (Cadena-Herrera et al. 2015).

The present findings should also be interpreted in the context of the wider MSC translation literature. Although the included studies reported promising proof-of-concept performance, substantial manufacturing barriers remain, including donor-to-donor and tissue-source variability, culture-induced senescence or phenotypic drift, inconsistent media and expansion protocols, scale-up challenges, and the absence of universally accepted potency assays. These factors can alter MSC biological activity and may limit the generalisability of AI models trained on single-site datasets. AI may help address some of these barriers by enabling longitudinal, non-destructive monitoring of culture morphology and quality; however, it should complement rather than replace functional testing. This need for robust and scalable quality assessment is also evident in gene-modified MSC products, where manufacturing processes must preserve cell identity, viability, genetic modification, and therapeutic function during expansion. For example, scalable microcarrier-based manufacturing of gene-modified hMSCs has been investigated to support more reproducible production (Couto et al. 2023), while clinical translation of MSCTRAIL illustrates the need for rigorous product characterisation and potency assessment in ex vivo gene-modified MSC therapies (Graham et al. 2026). Together, these studies support the clinical relevance of developing validated AI tools that can link measurable culture features with functional potency and eventual therapeutic performance.

Heterogeneity was a central finding rather than simply a methodological limitation. Studies differed in MSC tissue source, donor characteristics, culture conditions, imaging modality, AI architecture, reference standards, and the biological outcomes being predicted. Consequently, an accuracy value reported for morphology-based classification cannot be interpreted as directly comparable with a Dice score for image segmentation, a correlation coefficient for continuous prediction, or an F1 score obtained from an imbalanced classification dataset. This variation likely explains the broad range of reported performance and precluded meaningful quantitative pooling. It also highlights the need for future studies to report datasets, class balance, reference standards, validation procedures, and a core set of task-appropriate performance measures in a consistent manner.

Our findings are consistent with the broader literature showing that automated image analysis can support cell detection, segmentation, and morphology-based characterisation while reducing reliance on manual assessment (Cadena-Herrera et al. 2015). Within the included literature, AI was applied across several distinct but complementary tasks, including identification of culture errors from morphology (Imai et al. 2018), cell segmentation and phenotype classification (Mota et al. 2021), prediction of multipotency across donors (Kim et al. 2022b), and identification of senescent cells from bright-field images (He et al. 2024). Collectively, these studies support the potential of AI to detect biologically relevant culture features. However, most models were developed using relatively limited, site-specific datasets and were evaluated against laboratory surrogate outcomes rather than clinical potency or therapeutic efficacy. The present review therefore suggests that AI-based MSC assessment is promising, but remains at a proof-of-concept stage rather than being ready for routine clinical manufacturing.

Limitations of the review include study heterogeneity which precluded meta-analysis, and reliance on English-language publications. Our search strategy did not include the terms ‘umbilical cord’ or ‘Wharton’s jelly’. As terminology for umbilical cord-derived MSCs is not fully standardised, relevant studies may have been missed despite the broader MSC search terms used. While accuracy, sensitivity and F1 were common, some studies used less standard metrics such as LOO-R^2^ or PCC, making comparisons difficult. Reported performance metrics varied substantially across studies owing to differences in AI models, imaging modalities, datasets, and outcome definitions. For example, in one study precision ranged from as low as 21.6% (Mota et al. 2021) which may have been due to imbalanced datasets aimed at detecting rare culture errors, to over 90% in CNN-based stemness predictions (Kim et al. 2022b). These wide ranges, driven by differences in AI models, imaging inputs, and outcome definitions, make direct comparisons across the studies difficult and further underscore the need for standardized evaluation protocols with consistent metrics.

Future research should focus on standardizing outcome reporting of AI models for MSCs (2024). Such research should consider practical and ethical issues related to data accessibility, privacy, governance, and ethical oversight. Many AI models require large, well-labelled datasets (Li et al. 2025), such that most stem cell labs may need to generate and label their own data. Ethically, using patient-derived cells would require strict adherence to consent and privacy. Dedicated studies are also needed for other stem cell types to address the current gap, for example Limbal Epithelial Stem Cells cultures could be assessed with AI given their central role in corneal regeneration. In addition, external validation of AI models for stem cells with independent datasets and the use of shared benchmarks will be critical for translating AI from lab-based models into clinically robust tools for regenerative medicine.

Importantly, most included studies represented early-stage proof-of-concept investigations rather than clinically validated AI systems, and therefore their reported performance should be interpreted cautiously.

## Conclusion

This systematic review found that AI shows promising potential for non-destructive assessment of mesenchymal stromal/stem cell culture characteristics, including morphology, confluence, senescence, viability, and stemness-related features. However, the evidence remains heterogeneous and largely limited to early proof-of-concept studies using small, site-specific datasets and laboratory surrogate outcomes. Before AI can be adopted in routine MSC manufacturing or clinical translation, models require standardised outcome definitions and reporting, independent external validation, and evaluation against meaningful measures of cell potency and therapeutic function. AI should therefore be viewed as a potentially valuable adjunct to, rather than a replacement for, established functional and quality-control testing.

## Supplementary information

Below is the link to the electronic supplementary material.

Supplementary file1 (DOCX 33 KB)

## Data Availability

No datasets were generated or analysed during the current study.

## Glossary

Artificial Intelligence (AI): A software system capable of performing tasks typically requiring human intelligence such as image recognition and data classification.
Mesenchymal stromal/stem cells (MSCs): Multipotent, plastic-adherent cells capable of self-renewal and differentiation into osteogenic, adipogenic, and chondrogenic lineages, and typically characterised by a defined pattern of cell-surface marker expression**.**
Limbal epithelial stem cells (LESCs): Stem cells located at the corneal limbus that maintain and regenerate the corneal epithelium throughout life.
Stemness: A term referring to the quality of a cell that indicates its ability to self-renew and differentiate or turn into other cell types.
Viability: A term referring to the health and survival of a cell often assessed through metabolic activity or membrane integrity
Neural Networks: A type of AI model inspired by the structure and connections of biological neurons.
Hybrid Models: AI systems that combine two or more algorithms or model architectures to perform a task.
Sensitivity: The ability of a test or model to correctly identify true positives.
Specificity: The ability of a test or model to correctly identify true negatives.
F1 Score: A metric that balances precision and recall to gauge accuracy.
AUC (Area Under the Curve): A performance metric indicating how well a model can distinguish between classes.
Accuracy: The proportion of correctly classified predictions among all predictions.
LOO-R^2^ score: A metric which predicts unseen data by leaving out one sample at a time during training
Precision: The ratio of true positives to all predicted positives.
Dice score coefficient: A metric that quantifies the overlap between two sets.
R value: A measure of the linear relationship between two variables.
PROBAST: A tool used to assess the risk of bias and applicability in prediction model studies.
