# Supplementary material for: Artificial intelligence for stromal cell quality assessment: A systematic review of human mesenchymal stromal cell properties and viability
Source: Cell Tissue Res. 2026 Oct 15;405(3):13. doi: 10.1007/s00441-026-04097-y (PMC13672381; doi:10.1007/s00441-026-04097-y)
Supplement: Supplementary file 1 [file 441_2026_4097_MOESM1_ESM.docx]

**Search Report**

**Artificial Intelligence for Assessing Stemness and Viability in Mesenchymal Stem Cell Cultures: A Systematic Review:**

Time frame: 3^rd^ of December 2024 to 11^th^ of April 2025

Searches by: William Li, Athanasia Vardakis, and Shrushti Surya

Search results sent: Sent to Covidence software on the 11^th^ of April

Results:	TOTAL: 3040

TOTAL after de-duplication: 2569

| Source | Version/Platform/url | Date of Search | RCT filter applied | Records retrieved |
| --- | --- | --- | --- | --- |
| 1. MEDLINE In-process and other non-indexed citations and MEDLINE 1946-present | (Ovid SP) | 3^rd^ March 2025 | No | 1270 |
|  |  |  |  |  |
| 2. EMBASE  1972-2016 week 3 | (Ovid SP) | 11^th^ April 2025 | No | 574 |
|  |  |  |  |  |
| 3. CENTRAL | The Cochrane Library | 2^nd^ February 2025 | No | 702 |
|  |  |  |  |  |
| 4. Clinicaltrials.gov | [www.clinicaltrials.gov](http://www.clinicaltrials.gov) | 3^rd^ January 2025 | No | 0 |
| TOTAL before de-duplication | | | | 3040 |
| TOTAL after de-duplication | | | | 2569 |

Appendix A. Search Strategies:

| **Database searched** | **Search terms used** | **Records retrieved** |
| --- | --- | --- |
| 1. MEDLINE In-process and other non-indexed citations and MEDLINE 1950-present (Ovid SP) | (Mesenchymal stem cells[Title]) AND (Stemness[Title])  (Mesenchymal stem cells[Title]) AND (Viability[Title])  (Limbal epithelial cell[Title])  ("artificial intelligence" OR "machine learning" OR "deep learning" OR "image analysis") AND ("mesenchymal”) | 117  98  20  807 |
| 2. EMBASE  1980- (Ovid SP) | ("artificial intelligence" OR "machine learning" OR "deep learning" OR "image analysis") AND ("mesenchymal”)  Mesenchymal stem cell AND viability (Title searches only)  Mesenchymal stem cell AND stemness (Title searches only)  Mesenchymal stem cells AND stemness (Title searches only)  (((Mesenchymal stem cell and stem cell and stemness) or viability) and AI and artificial intelligence).mp. [mp=title, abstract, heading word, drug trade name, original title, device manufacturer, drug manufacturer, device trade name, keyword heading word, floating subheading word, candidate term word] | 3014  50  104  154  227 |
| 3. CENTRAL (*The Cochrane Library*) (Issue( insert issue number here)) | ("artificial intelligence"[tiab]) AND ("mesenchymal stem cells"[tiab] OR "cell culture"[tiab] OR "limbal epithelial cells"[tiab]) | 47 |
| 4. Clinicaltrials.gov ([www.clinicaltrials.gov](http://www.clinicaltrials.gov)) | Mesenchymal stem cells AND artificial intelligence  Mesenchymal stem cells AND Stemness | 0  0 |
| TOTAL retrieved | | 3040 |
| TOTAL after duplications were removed | | 2569 |
